# Development of Wafer-Type Plasma Monitoring Sensor with Automated Robot Arm Transfer Capability for Two-Dimensional In Situ Processing Plasma Diagnosis

**DOI:** 10.3390/s24061786

**Published:** 2024-03-10

**Authors:** Haewook Park, Juhyun Kim, Sungwon Cho, Kyunghyun Kim, Sungho Jang, Younsok Choi, Hohyun Lee

**Affiliations:** Mechatronics Research, Samsung Electronics Co., Ltd., 1-1, Samsungjeonja-ro, Hwaseong-si 18448, Gyeonggi-do, Republic of Korea; hw92.park@samsung.com (H.P.); juhyun92.kim@samsung.com (J.K.); sungwon5.cho@samsung.com (S.C.); krrg.kim@samsung.com (K.K.); tiger77.jang@samsung.com (S.J.)

**Keywords:** semiconductor manufacturing, processing chamber, wafer-type sensor, in situ, plasma diagnosis, plasma density, tool-to-tool matching (TTTM)

## Abstract

In this work, we propose our newly developed wafer-type plasma monitoring sensor based on a floating-type double probe method that can be useful for two-dimensional (2D) in situ plasma diagnosis within a semiconductor processing chamber. A key achievement of this work is the first realization of an ultra-thin plasma monitoring sensor with a system thickness of ~1.4 mm, which supports a fully automated robot arm transfer capability for in situ plasma diagnosis. To the best of our knowledge, it is the thinnest accomplishment among all wafer-type plasma monitoring sensors. Our proposed sensor is assembled with two Si wafers and SiO_2_-based probes; accordingly, it makes it possible to monitor the actual dynamics of processing plasmas under electrostatic chucking (ESC) conditions. Also, it allows for the prevention of chamber contamination issues after continuously exposing the radio frequency (RF) to various processing gases. Using a test-bed chamber, we successfully demonstrated the feasibility and system performance of the proposed sensor, including robot arm transfer capability, vacuum and thermal stress durability, and data integrity and reproducibility. Consequently, compared with the conventional plasma diagnostic tools, we expect that our proposed sensor will be highly beneficial for tool-to-tool matching (TTTM) and/or for studying various plasma-related items by more accurately providing the parameters of processing plasmas, further saving both time and manpower resources required for preventive maintenance (PM) routines as well.

## 1. Introduction

Low-temperature plasmas have been actively investigated in many academic research studies and industrial fields [1,2,3,4,5]. Especially in semiconductor manufacturing, it is important to carefully monitor the dynamics of low-temperature plasmas within a processing chamber to increase semiconductor yield. For this reason, many different plasma diagnostic tools have been studied and widely used to unveil the nature of plasmas [6,7,8,9,10,11,12,13,14].

Among them, one of the most commonly used plasma diagnostic tools in semiconductor processing is a needle-type probe—specifically, the Langmuir probe is the traditional and representative invention for in situ plasma monitoring [15,16]. These needle-type probes still play a crucial role in exploring various plasma-related study items and are also widely used for a tool-to-tool matching (TTTM) procedure during a preventive maintenance (PM) routine of the processing chamber.

However, there are several drawbacks to utilizing needle-type plasma diagnostic tools. First, it may lead to a huge discrepancy in plasma parameters in comparison to those under a real processing condition. This is because the vertical position of a Langmuir probe is very distant from the electrostatic chuck (ESC) surface (i.e., ~few centimeters) where a real production wafer is located. Second, it is barely possible to measure two-dimensional (2D) plasma distribution because a needle-type probe can provide only single-channel data per scan. Even a horizontal (or vertical) step-wise scan requires additional human interactions to modify the probe position. Third, it is a “wired” system, so inserting a needle-type probe through a viewport of a processing chamber is too laborious and time-consuming, especially owing to the narrow spacing between the semiconductor equipment in a real production line.

To address the aforementioned issues, there have been many attempts to monitor various plasma parameters by means of developing wafer-type plasma sensors. Lim et al. [17] proposed a wafer-type plasma sensor that is capable of simultaneously measuring the 2D distribution of plasma parameters. However, the sensor is not compatible in size with a 12-inch chamber. Also, it is not suitable for automated semiconductor processing because its system thickness exceeds 1.6 mm (i.e., the maximum height of the integrated circuits (ICs) utilized in the sensor exceeds 1.6 mm), which is beyond the height limit in typical semiconductor equipment during the automated robot arm transfer. Similarly, Han et al. [18] implemented a wafer-type ion energy monitoring apparatus for in situ monitoring of the semiconductor process; likewise, it can only provide plasma information over a 6-inch processing chamber. Meanwhile, Kim et al. [19] fabricated a wireless wafer-type plasma probing system that can be useful for a 12-inch processing chamber; however, its system thickness is still too thick (i.e., 12 mm) to be used for industrial applications, and the aluminum-based materials are not compatible for semiconductor processing.

Hence, in this study, we propose a newly developed wafer-type plasma monitoring sensor with an ultra-thin system thickness of ~1.4 mm, which supports a fully automated robot arm transfer capability in semiconductor equipment for in situ plasma diagnosis. To the best of our knowledge, it is the first and thinnest realization among all wafer-type plasma diagnostic tools. Thanks to its ultra-thin structure, our solution takes advantage of performing the plasma diagnosis across multiple processing chambers easily and efficiently, by simply manipulating a cluster tool controller (CTC) with no requirement for additional human interactions. Our proposed sensor is assembled with silicon-based materials, thereby allowing it to monitor the actual dynamics of processing plasmas even under electrostatic chucking (ESC) conditions. Additionally, the proposed sensor can be repeatedly mounted on the same location of the ESC surface via a robot arm transfer function, and it could provide superior system performance in terms of high data reproducibility and reliability. Therefore, we can expect that the resulting plasma parameters would be more highly correlated with the real outputs (i.e., etch rate distribution) from semiconductor processing.

## 2. Materials and Methods

### 2.1. Double Probe Based on Floating Harmonic Method

In the proposed sensor, we employed a floating harmonic method [20,21,22], which is one of the most practical solutions for the in situ monitoring of processing plasmas because it is hardly affected by the deposition on probe tips during the deposition and etching process.

**Figure 1:**
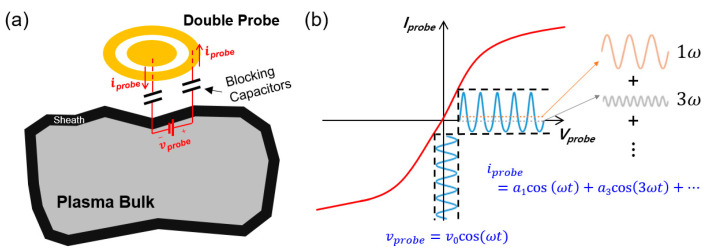
(**a**) Conceptual diagram of a double probe based on a floating harmonic method used for plasma diagnosis in this development. (**b**) I-V characteristics of the double probe at a floating potential.

Specifically, we adopted a double probe configuration since it allows for the differential signaling of plasma current and enables our proposed sensor to be electrically floated with the ground of the processing chamber [23]. In principle, the double probe based on a floating harmonic method measures the electrical current incoming from processing plasmas at a floating potential, after forcing the low-frequency sinusoidal voltage into between two probes, as described in Figure 1a.

Note that, unlike the conventional Langmuir probe with a cylindrical design, our double probe design restricts the full-range measurement of electron energy distribution functions (EEDFs) due to its inability to apply external voltages that exceed the plasma potential on the double probe [24,25]. Therefore, the distribution of these electrons collected by the proposed sensor may not be representative of the distribution of electrons in the plasma bulk [8].

Let us define the ion currents to the inner probe and outer probe as $i_{ion\_in}$ and $i_{ion\_out}$, and the electron current to the inner probe and outer probe as $i_{e\_in}$ and $i_{e\_out}$, respectively. Assuming the double probe is operated at a floating potential, no net current flows from the double probe into the plasma, as shown in Equation (1).

(1) $$i_{ion\_in}+i_{ion\_out}-i_{e_{in}}-i_{e\_out}=0$$

Also, the local probe current ($i_{probe}$) forming a closed loop between the double probe and the plasma can be represented as Equation (2).

(2) $$i_{probe}≝i_{e\_in}-i_{ion\_in}=i_{e\_out}-i_{ion\_out}$$

Assuming that the electrons entering the double probe follow a Maxwellian distribution, the electron current to the each of inner and outer probes can be delineated as Equations (3) and (4) where $A_{in}$ is the sensitive area of the inner probe and $A_{out}$ is the sensitive area of the outer probe, $J_{esat\_in}$ is the electron saturation current density of the inner probe, $J_{esat\_out}$ is the electron saturation current density of the outer probe, $v_{in}$ is the voltage of inner probe with respect to the plasma potential, $v_{out}$ is the voltage of the inner probe with respect to the plasma potential, and $T_{e}$ is the electron temperature measured in the vicinity of the double probe.

(3) $$i_{e\_in}=A_{in}\cdot J_{esat\_in}\cdot \exp(v_{in}/T_{e})$$

(4) $$i_{e\_out}=A_{out}\cdot J_{esat\_out}\cdot \exp(v_{out}/T_{e})$$

Subsequently, Equation (2) can be rearranged as Equation (5).

(5) $$\frac{{i_{ion\_in}+i}_{probe}}{{i_{ion\_out}-i}_{probe}}=\frac{A_{in}}{A_{out}}\cdot \exp((v_{in}-v_{out})/T_{e})$$

When the sensitive areas of both inner and outer probes are identical ($A_{in}=A_{out})$, Equation (5) can be transformed in terms of probe current ($i_{probe}$), as shown in Equation (6) where $v_{probe}$ is the differential bias voltage ($v_{in}-v_{out})$ across the inner and outer probes and $i_{ion}$ is the net ion current flowing through the inner and outer probes ($i_{ion\_in}-i_{ion\_out})$.

(6) $${i_{probe}=i}_{ion}\cdot \tanh\left(v_{probe}/2T_{e}\right)$$

As the plasma is ignited, the probe current ($i_{probe}$) features the nonlinear I-V curve characteristics as the small sinusoidal bias voltage ($v_{probe}=v_{0}\cos\omega t)$ is introduced into the double probe (Figure 1b). In this situation, the probe current expressed in Equation (6) can be represented as Equation (7) by substituting the term $v_{probe}/2T_{e}$ into αcos⁡ωt where we denote α as a constant value of $v_{0}/2T_{e}$ [19,23].

(7) $$i_{probe}=i_{ion}\cdot \tanh\left(\alpha \cos\omega t\right)\;s.t.\;\alpha ≝v_{0}/2T_{e}$$

Since the $\tanh\left(\cdot \right)$ is an odd function, we could separate the probe current using odd harmonic components based on a Taylor series expansion. Here, if the amplitude of the injected sinusoidal wave ($v_{0}$) is small enough for the electron temperature ($T_{e}$), Equation (1) approximately becomes the function of fundamental frequency (1ω) and third harmonic component (3ω), as described in Equation (8) [19,23].

(8) $$\begin{array}{l} i_{probe}\\ =i_{ion}\cdot \left(\alpha \cos\omega t-\frac{1}{3}\alpha^{3}\cos^{3}\omega t+\frac{2}{15}\alpha^{5}\cos^{5}\omega t+\cdots \right),\\ \approx i_{ion}\cdot \left\{\left(\alpha -\frac{\alpha^{3}}{4}\right)\cos\omega t+\left(-\frac{\alpha^{3}}{12}\right)\cos3\omega t\right\} \end{array}$$

Now we see that the amplitude of each harmonic frequency component in Equation (8) consists of the factor of α. For the sake of simplicity, let us denote the term amplitude ratio (R), as shown in Equation (9). We subsequently can express the α in terms of the amplitude ratio (R), as shown in Equation (10) [19,23].

(9) $$R≝\frac{\left|I_{probe\_3\omega }\right|}{\left|I_{probe\_1\omega }\right|}=\frac{\left|-\frac{\alpha^{3}}{12}\right|}{\left|\alpha -\frac{\alpha^{3}}{4}\right|}=\frac{\alpha^{2}}{\left|12-3\alpha^{2}\right|}\;$$

(10) $$\alpha =\sqrt{\frac{12R}{3R+1}\;}$$

Consequently, if we know the amplitude of differential bias voltage ($v_{0}$) and the amplitude of both fundamental ($\left|i_{probe\_1\omega }\right|$) and third harmonic ($\left|i_{probe\_3\omega }\right|$) components of the probe current, by using the amplitude ratio (R) and the definition of constant α (Equations (9) and (10)), we can simply determine the electron temperature ($T_{e}$), as described in Equation (11) [19,23].

(11) $$\;T_{e}=v_{0}/2\alpha =\sqrt{\frac{3R+1}{48R}}\cdot v_{0}$$

Subsequently, we can determine the ion current ($i_{ion}$) by simply using the amplitude of fundamental components ($\left|i_{probe\_1\omega }\right|$) of the probe current, as described in Equation (12) [19,23].

(12) $$i_{ion}\approx \frac{{|I}_{probe\_1\omega }|}{\left(\alpha -\frac{\alpha^{3}}{4}\right)}$$

If the processing plasma is composed of monoatomic gas (e.g., Ar, Cl_2_, N_2_, etc.), we can derive the plasma density by measuring the ion density ($n_{ion}$) based on the ion current ($i_{ion}$) and a Bohm velocity ($u_{Bohm}$), as shown in Equation (13) where A is the sensitive area of the double probe and e corresponds to the elementary charge constant with the value of 1.6 × 10^−19^.

(13) $$n_{ion}=\frac{i_{ion}}{0.61\cdot e\cdot u_{Bohm}\cdot A}$$

Assuming that the shape of the sheath on the probe surface is ideally planar, the ion flux ($\Gamma_{ion}$) can be simply calculated by dividing the ion current ($i_{ion}$) by the multiplication of elementary charge (e) and the probe area (A), as described in Equation (14).

(14) $$\Gamma_{ion}=\frac{i_{ion}}{e\cdot A}\;$$

Also, it is worth noting that the fundamental components ($\left|i_{probe\_1\omega }\right|$) of the probe current and the ion flux ($\Gamma_{ion}$) can be a surrogate solution of the plasma density when the plasma is produced based on processing gases consisting of different type of ion species (e.g., CF4, HBr, etc.).

### 2.2. Wafer-Type Plasma Monitoring Sensor

#### 2.2.1. System Characteristics

**Figure 2:**
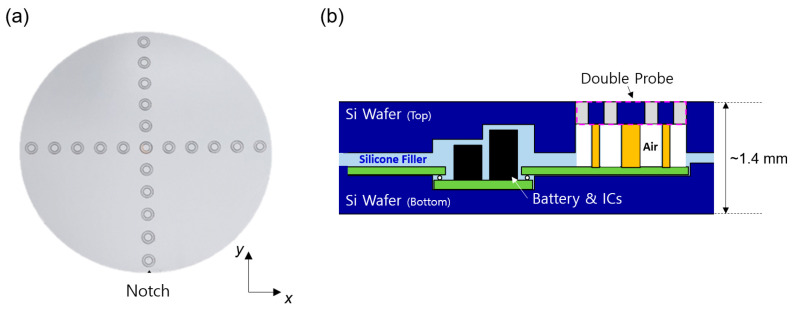
(**a**) Photo of the proposed wafer-type monitoring sensor. (**b**) Conceptual cross-sectional diagram of the proposed sensor.

Figure 2a presents the photo of the proposed wafer-type plasma monitoring sensor. The notch part in the proposed sensor is designed to be the same as a real production wafer, hence it is compatible with a pre-aligner module within the equipment front-end module (EFEM). Our proposed sensor consists of SiO_2_-based double probes (21 ea) distributed in each x- and y- direction, radio frequency (RF) choke filters, rechargeable lithium (Li)-ion batteries, discrete analog and digital ICs, and a receiver (Rx) coil for wireless charging. All the sensor components were coated with a polymer to enhance electrical insulation with adjacent Si wafers (i.e., each of the top and bottom Si wafers) before the full assembly. We embedded a temperature sensor IC within the proposed sensor for auxiliary temperature monitoring.

Figure 2b shows the conceptual cross-sectional diagram of the proposed sensor. The system thickness of the sensor prototype is ~1.4mm. Each of the components, except for double probes, is internally buried between the top and bottom Si wafers. In order to precisely monitor the plasma dynamics experienced by the wafer surface during actual semiconductor processing, it is advantageous to fabricate the wafer-type sensor with a thickness similar to that of the real production wafer (i.e., 775 μm). However, there exists a trade-off between the thickness and the structural integrity of the sensor assembly as it becomes more susceptible to cracking due to temperature changes and the electrostatic chucking process. The mechanical process (i.e., etching and grinding) of the Si wafer was carefully performed and optimized via experiments to achieve both good heat tolerance and mechanical durability at an ESC temperature of 70 °C under ESC chucking conditions.

Considering the warpage issue owing to the thermal expansion of a Si wafer, a silicone filler and Si-based adhesive materials were applied between the two Si wafers. After this process was complete, the sample was left under high vacuum conditions for an extended period of time to prevent particle issues caused by outgassing from adhesive materials and to further prevent the Li-ion battery from swelling.

**Figure 3:**
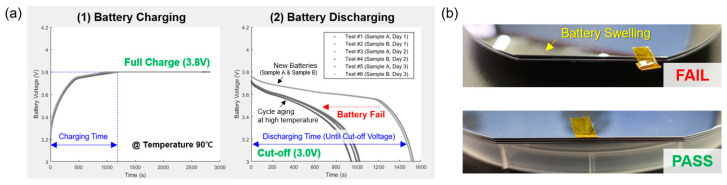
Experimental assessment of a Li-ion battery in terms of vacuum resistance and heat tolerance. (**a**) Example results of a battery aging after the long-run heat tolerance test while repeatedly charging and discharging the Li-ion battery under a high temperature of 90 °C. (**b**) Test samples: a failed case (i.e., due to battery swelling) and a passed case during the vacuum resistance test.

In order to further understand the performance of a Li-ion battery under a harsh environment, we performed an experiment using different wafer conditions (Figure 3). The vacuum condition was <1 mTorr and the temperature was set to 90 °C. Throughout the above experiments, we could optimize several parameters for sensor assembly (i.e., amount of silicone fillers) that yield good mechanical durability and a long battery lifetime performance of the proposed sensor under a high-vacuum and a high-temperature condition. We have noted that the operating temperature of the battery and ICs used in our wafer-type sensor guarantee its operation until the lower temperature limit of −20 °C and −40 °C, respectively; therefore, we expect that our wafer-type sensor even can work properly under a low-temperature semiconductor processing condition. However, the impact of the low temperature on diagnostic performance is beyond the scope of this study and requires further investigation.

The main features of the proposed wafer-type plasma monitoring sensor are summarized below:

- Twelve-inch processing chamber compatibility;
- Floating-type double probe-based plasma diagnosis tool;
- Automated robot arm transfer capability via CTC software;
- Si- and SiO_2_-based materials with ultra-thin system design (i.e., thickness ~1.4 mm, weight ~210 g);
- Microprocessor unit (MCU)-based embedded system;
- Rule-based data acquisition using in-house designed software;
- Rechargeable Li-ion battery-based wireless system;
- Vacuum resistance ≤1 mTorr;
- Operation temperature −20 °C to 70 °C;
- Bias RF ≤500 W, Source RF ≤2500 W @ ICP chamber.

#### 2.2.2. Probe Design

**Figure 4:**
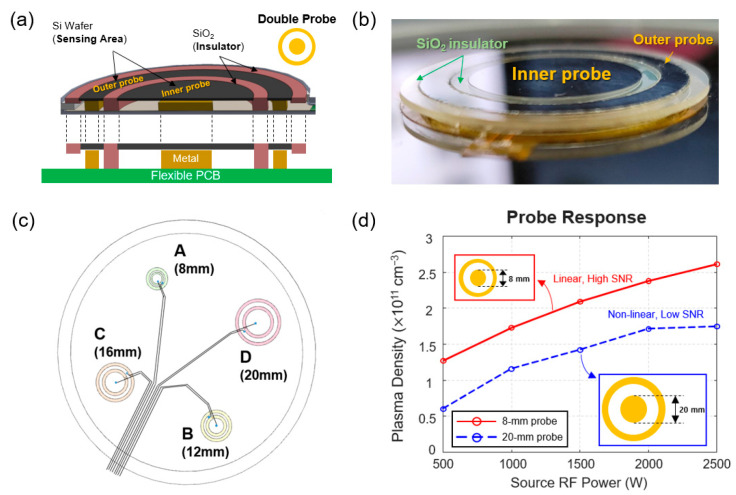
(**a**) Cross-sectional view and the structure of a double probe used in this work. (**b**) Photo of the double probe. (**c**) Four candidates of the probe design in terms of different sensitive areas of the inner probe (i.e., 8 mm, 12 mm, 16 mm, and 20 mm). (**d**) Experimental results of the two double probe responses (i.e., 8-mm (smallest) and 20-mm (largest) probe units) with increasing source RF power in steps of 500 W.

Figure 4 shows the probe design of the proposed wafer-type plasma monitoring sensor. As shown in Figure 4a,b, we designed the double probe that features a concentric circle structure based on a Si wafer and SiO_2_-based materials to prevent chamber contamination issues after continuously exposing the radio frequency (RF) to various processing gases. The spacing between the inner and outer probes was carefully designed to be sufficient to prevent undesirable leakage currents. This concentric circle structure can mitigate the spatial error of plasma parameters during the sensor operation. The probe area, which is sensitive to processing plasmas (i.e., the black area shown in Figure 4a), for each of the inner and outer probes was designed to be identical. We experimentally selected the optimal size of the double probe that can yield the plasma parameters with a high correlation to the theoretical values. A total of four types of probe designs were investigated with varying the diameter of the inner probe (Figure 4c). Throughout the experiment (Figure 4d), we selected the double probe design with an inner probe diameter of 8 mm because it showed the optimal performance among other probe units in terms of linearity and signal-to-noise ratio (SNR) performance under a 500 W to 2500 W source RF sweep condition. This result may infer that the oversized probe units may noticeably perturb the plasmas during the diagnosis. We note that it is necessary to consider the collision of electrons with the probe units in the plasma diagnosis. Considering that the mean free path of electrons (i.e., approximately 25~30 mm @ 20 mTorr) [8,26,27] is sufficiently longer than the dimension of probe radius (i.e., 8 mm), we conjecture that only a small amount of collisions of electrons will affect our flat-type double probe, thereby yielding no significant disturbances during plasma diagnosis. To secure a high level of probe flatness, we introduced a novel SiO_2_-reflow process into the probe assembly. The double probe is finally assembled into the proposed system via a metal structure and flexible printed circuit board (PCB).

#### 2.2.3. Operation Circuit for Plasma Diagnosis

**Figure 5:**
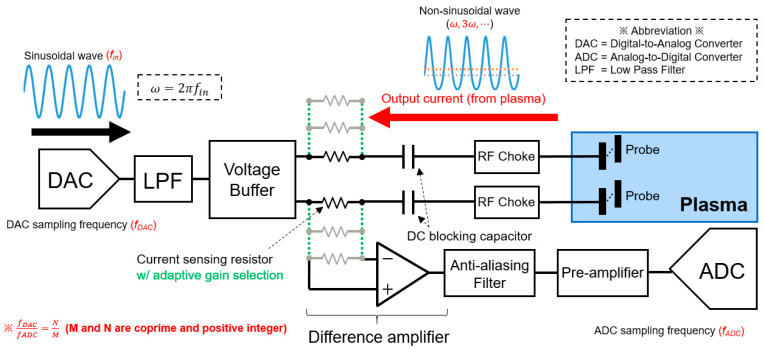
Simplified circuit diagram for plasma diagnosis of the proposed wafer-type plasma monitoring sensor.

Figure 5 shows the operation circuit for plasma diagnosis embedded within the proposed sensor. It consists of several modules that include a digital-to-analog converter (DAC), a low-pass filter (LPF), a voltage buffer, a current sensing resistor, a DC blocking capacitor, a custom-designed RF choke filter, a difference amplifier, an anti-aliasing filter, a preamplifier, and an analog-to-digital converter (ADC).

For signal generation, we used the DAC with a 12-bit resolution and a 1024-kSPS sampling rate. It generates a low-frequency sinusoidal wave of 8 kHz. The DAC parameters, such as amplitude and operation frequency, are fully adjustable by using an in-house designed data acquisition software (Section 2.2.5). The operation frequency was carefully chosen to generate less perturbation to processing plasmas. The DAC output is then connected to the double probe after coarsely eliminating the high-frequency noise through the passive LPF with a cutoff frequency of 10kHz and followed by the voltage buffer module. The voltage buffer module was designed using a general-purpose operational amplifier with a unity gain.

When the plasma is ignited after biasing the double probe, the probe current with harmonic components (i.e., $i_{probe}$ depicted in Figure 1a) begins flowing toward the current sensing resistor via the DC blocking capacitor. Subsequently, the probe current is converted into the voltage signal via the current sensing resistor and the difference amplifier. Here, we adopted an adaptive gain variation technique that enables us to choose an optimal current sensing resistor value suitable for the plasma environment. The custom-designed RF choke filter was tuned and applied to attenuate both 13.56-MHz and 27.12-MHz (i.e., second harmonics) RF interferences from an RF generator of semiconductor equipment.

Considering the Nyquist sampling theorem, we added an anti-aliasing filter that removes the undesirable aliasing from the converted voltage signal trespassed from other frequency bands. Here, we have adopted the 3rd-order analog Chebyshev filter to achieve a steeper roll-off than the conventional Butterworth filter. The low-bandwidth preamplifier was employed to control the DC level and the gain of the ADC input signal that allows for the use of a full dynamic range of the ADC. Finally, the converted voltage signal is sampled with the ADC module with a vertical resolution of 12 bits and a sampling rate of 256 kSPS. The ADC samples then undergo a fast Fourier transform (FFT) process to perform data analysis in the frequency domain. All the data were then tentatively stored in nonvolatile memory and wirelessly transferred to a personal computer (PC) via a sensor carrier platform (Section 2.2.4) that allows for on-demand data analysis to the user.

**Figure 6:**
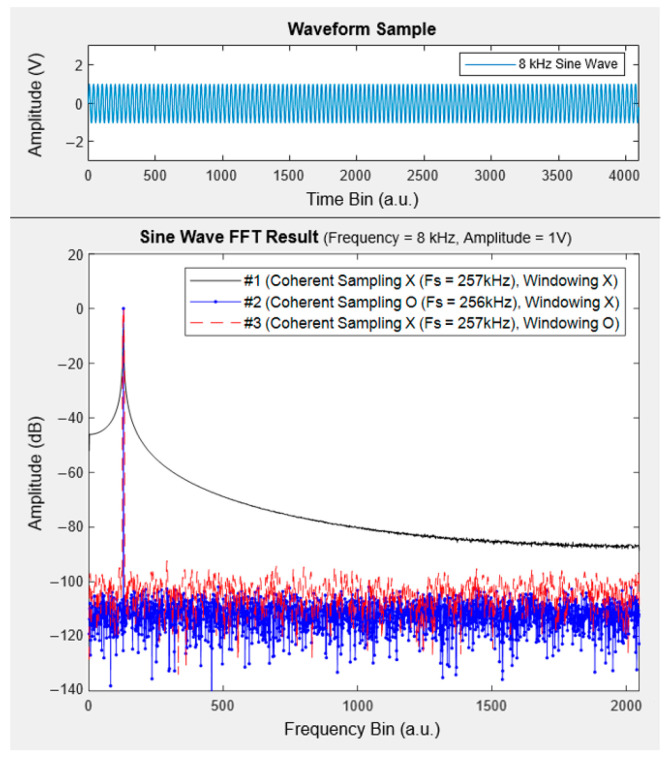
Effect of coherent sampling and/or window function (i.e., flat-top window, Hann window, Hamming window, etc.) in FFT results. Case #1—No coherent sampling and/or window function is applied. Case #2—Only the coherent sampling is applied. Case #3—Only the window function is applied.

One major bottleneck in implementing ultra-thin wafer-type plasma monitoring sensors based on a floating harmonic method is the power consumption and computational burdens during the analog and digital signal processing of probe currents. To overcome the aforementioned issues, we employed a coherent sampling (and/or window function) technique—which is one of the key breakthroughs in this development—so that the ratio between the signal frequency and the sampling frequency was set to be a co-prime number and a number of FFT sampling bin as a power of 2. It allowed for reducing the sampling speed of the ADC by a factor of 64, while the previous study utilized the ADC with a 16-MSPS sampling rate [19]. Figure 6 shows the effect of coherent sampling and/or window function, which significantly reduces the undesirable spectral leakage in the frequency domain and lowers the noise floor of ADC using a small number of FFT samples. Therefore, it becomes easier to detect the plasma signals with an excellent SNR even under a noisy and harsh plasma condition. Moreover, we incorporated several innovative algorithmic techniques during the digital signal processing—one example corresponds to the technique that only records the peak amplitudes with a frequency-of-interest among the full FFT data (i.e., not all FFT samples in the frequency domain). It dramatically reduces the data storage and saves the computation burden, thus guaranteeing a much longer lifetime of sensor usage.

Along with the above implementation, the overall power consumption of the proposed system was 50 times less than that of the conventional wafer-type plasma monitoring system [19], which subsequently made it possible to operate the proposed sensor using a Li-ion battery with low capacity.

#### 2.2.4. Sensor Carrier Platform

**Figure 7:**
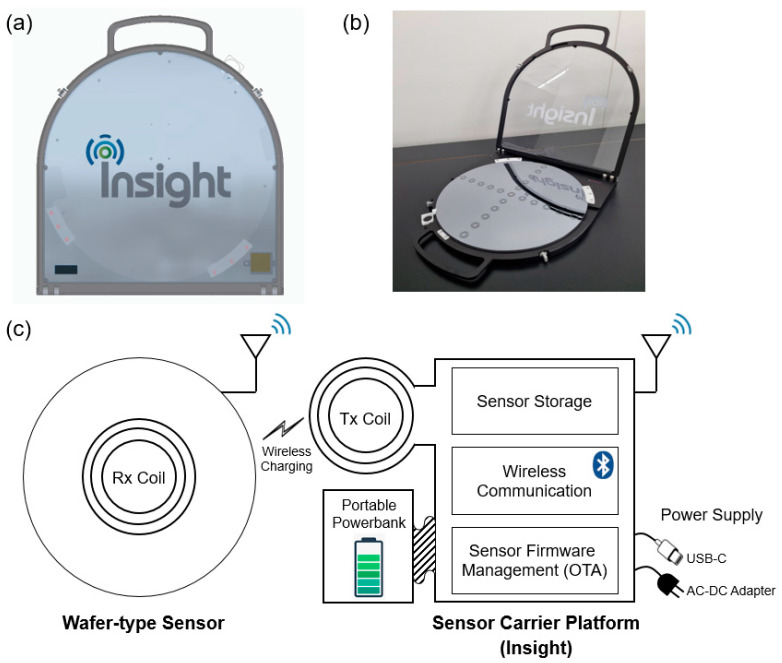
(**a**) The 3D rendered image and (**b**) photo of the sensor carrier platform that is compatible with other wafer-type sensors. (**c**) Schematic diagram and the functionality of sensor carrier platform.

In order to effectively manage the wafer-type sensor products, we custom-designed a sensor carrier platform (i.e., we named it “Insight”), as shown in Figure 7a,b. The exterior part of the sensor carrier platform is made of aluminum and polycarbonate, and the interior part is made of a polyacetal material with antistatic coating. In an aspect of versatility, we designed the sensor carrier platform to be compatible with all other types of wafer-type sensors that we have (or will be) developed. In this work, we have practically utilized the sensor carrier platform during the feasibility assessment and the live demo test at a real production line.

Figure 7c graphically describes a function of the sensor carrier platform, which includes (1) sensor storage; (2) wireless charging (transmission coil; Tx coil); (3) wireless communication with a wafer-type sensor via Bluetooth low energy (BLE) protocol; (4) sensor firmware management via over-the-air (OTA) programming; (5) auxiliary power bank for portable use (e.g., for a real production line application); (6) universal serial bus (USB, C-type) compatibility for serial communication with the sensor and auxiliary power supply; (7) 220 V power supply via a universal AC-DC power adapter. Thanks to our in-house-developed sensor carrier platform that features wireless battery charging and a remote data communication capability, we expect that our proposed wafer-type sensor can be seamlessly integrated with existing semiconductor manufacturing processes using the data server installed in semiconductor production lines.

#### 2.2.5. Data Acquisition Software

**Figure 8:**
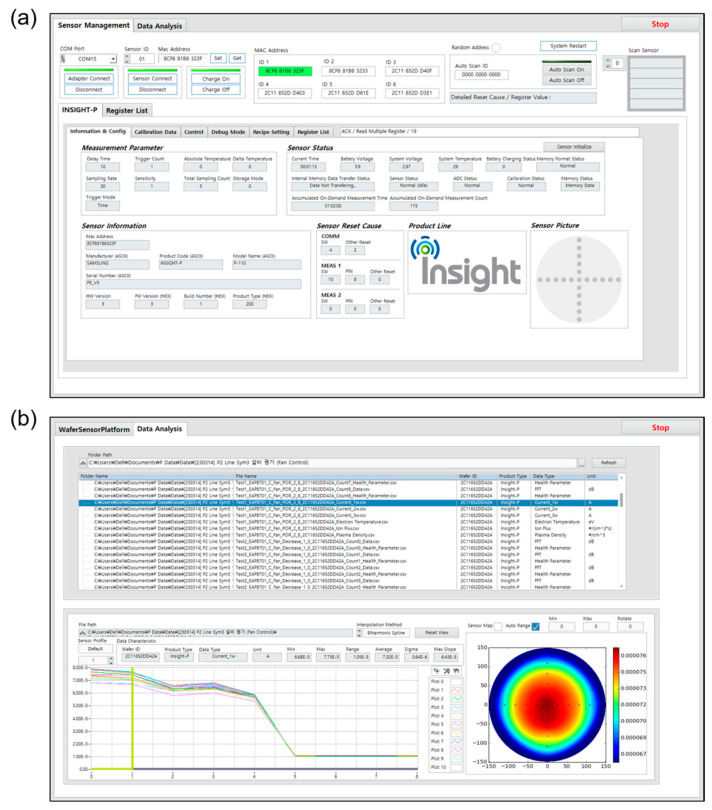
In-house-designed data acquisition software. (**a**) Sensor Management tab: a console window for sensor management; (**b**) Data Analysis tab: a display window to examine and analyze the results of plasma diagnosis on an event-by-event basis.

Also, we successfully developed data acquisition software for the proposed sensor based on a LabVIEW development environment, as shown in Figure 8. The proposed sensor can be connected to the data acquisition software via Bluetooth low energy (BLE) protocol. The proposed sensor can access the data acquisition software via the sensor carrier platform by mounting the proposed sensor onto the sensor carrier platform (Figure 7b). Figure 8 shows the user interface of our data acquisition software. To provide end-users with a more familiar experience, we designed the function and interface of our rule-based data acquisition software to resemble that of the existing software. In addition, our rule-based data acquisition software supports a unique plasma-driven data acquisition mode. Typically, the conventional rule-based data acquisition systems rely on time delays to trigger the measurement, which may be inefficient in plasma diagnosis due to variations in plasma ignition times across different chambers. On the other hand, our plasma-driven data acquisition mode ensures that data sampling occurs only after the plasma has fully ignited, thereby minimizing measurement errors.

The developed data acquisition software consists of two main tabs.

(1) Sensor Management tab (Figure 8a): it is a console window that features several functions for sensor management, including sensor identification (i.e., version and type of a wafer-type sensor), sensor status alarm (i.e., normal or abnormal status), sensor parameter setup (i.e., number of FFT samples, DAC amplitude, etc.), operation mode selection (i.e., self-test mode, plasma diagnosis mode, calibration mode, etc.), and data storage (i.e., setup a file name and its save path).
(2) Data Analysis tab (Figure 8b): it is a display window for data analysis after the plasma diagnosis. Users can freely access the plasma information that includes raw signals, FFT data, and calculated plasma parameters (i.e., electron temperature, plasma density, and ion flux) on an event-by-event basis. For users’ convenience, it also provides a wafer-shaped 2D plot, and the users can adjust a data range, color bar setting, and interpolation options for the 2D display of plasma distribution. Especially, it enables users to create of a pop-up window and is, thus, suitable for comparatively analyzing multiple data at a glance.

## 3. Experimental Results

We experimentally demonstrated the feasibility and system performance of the proposed sensor using an inductively coupled plasma (ICP) test-bed chamber (MC-4, SEMES) after the full sensor assembly. For the ICP test-bed chamber, the operation frequency of the source and bias RF generator was 13.56 MHz. All the experiments were performed under a vacuum condition of 20 mTorr. Here, we note that in situ dry (ISD) cleaning was performed after each sensor evaluation to further avoid both chamber and sensor contamination issues.

### 3.1. Robot Arm Transfer Capability

First, we checked the robot arm transfer capability of the proposed sensor. Before the manipulation of CTC software, we inserted the proposed sensor into a front-opening unified pod (FOUP) and subsequently mounted it on a load port. 

As a result, thanks to the ultra-thin system thickness of ~1.4 mm and light weight of ~210 g, the proposed sensor was successfully loaded into the processing chamber via a robot arm installed at EFEM, after passing through each aligner module, the load-lock module, and the transfer module without any mapping errors, movement failure, sensor crack, and/or error messages on the CTC software. It is worth noting that our sensor prototype has completed the automated robot arm transfer function more than 350 times without any failures.

### 3.2. Thermal Stress Durability

**Figure 9:**
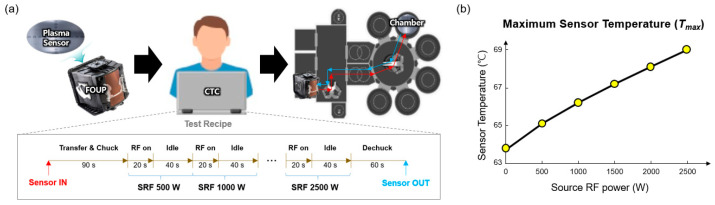
(**a**) Illustration of automated robot arm transfer scenario using the proposed sensor via a CTC for plasma diagnosis; here, we used a test recipe under a source RF (SRF) exposure condition with continuous wave (CW) mode by increasing the SRF power from 500 W to 2500 W in steps of 500 W using an argon gas. The source RF was irradiated once every 60 s per each step with an RF exposure time of 20 s and an idle time of 40 s. The vacuum condition was 20 mTorr. (**b**) Maximum temperature ($T_{max}$) recorded from the proposed sensor during the plasma diagnosis based on the test recipe where the ESC temperature of the processing chamber was 70 °C.

Second, we assessed the mechanical stability of the proposed sensor under an initial ESC temperature condition of 70 °C. We continuously exposed source RF (SRF) power of 3000 W using a continuous wave (CW) mode. We injected an argon (Ar) gas of 200 sccm into the processing chamber. We designed a test recipe for sensor evaluation that takes a total runtime of 532 s per a single test (Figure 9a), including a set of RF exposures, chuck and dechuck, and a robot arm transfer process. Here, we measured the sensor temperature using the temperature IC embedded within the proposed sensor, as delineated in Section 2.2.1.

Based on the results, the proposed sensor experimentally demonstrated its mechanical robustness without showing any sensor damage (i.e., arcing or crack) and/or chamber contamination issues. The maximum sensor temperature was recorded as less than 70 ℃ (Figure 9b) under an ESC temperature of 70 ℃ and a high SRF exposure condition of 2500 W. Also, most importantly, the proposed sensor did not show any significant functional abnormalities during the repetitive plasma diagnosis.

To investigate the sensor lifespan, we have performed (also ongoing) a long-term experiment; consequently, our proposed sensor has remained unaffected by discernible problems even after an accumulated RF exposure time of more than 5.5 h, which guarantees the proper operation of the sensor more than 350 times under a normal argon plasma condition (i.e., similar recipes described in Figure 9). The expected lifetime of the proposed sensor using processing gases is under investigation.

### 3.3. Data Integrity and Reproducibility

Finally, we evaluated the data integrity and reproducibility of the proposed sensor during the plasma diagnosis. Here, we measured each plasma density, ion flux, and electron temperature using an argon gas of 200 sccm, respectively. For the assessment of data integrity, we increased the SRF power from 500 W to 2500 W in steps of 500 W (i.e., 500 W→1000 W→1500 W→2000 W→2500 W), and for the assessment of data reproducibility, we repeatedly exposed the same SRF power of 1000 W during the measurement (i.e., 1000 W→1000 W→1000 W→1000 W→1000 W). During the evaluation of data integrity and reproducibility, we used the same chamber condition and recipe format, as depicted in Figure 9a.

**Figure 10:**
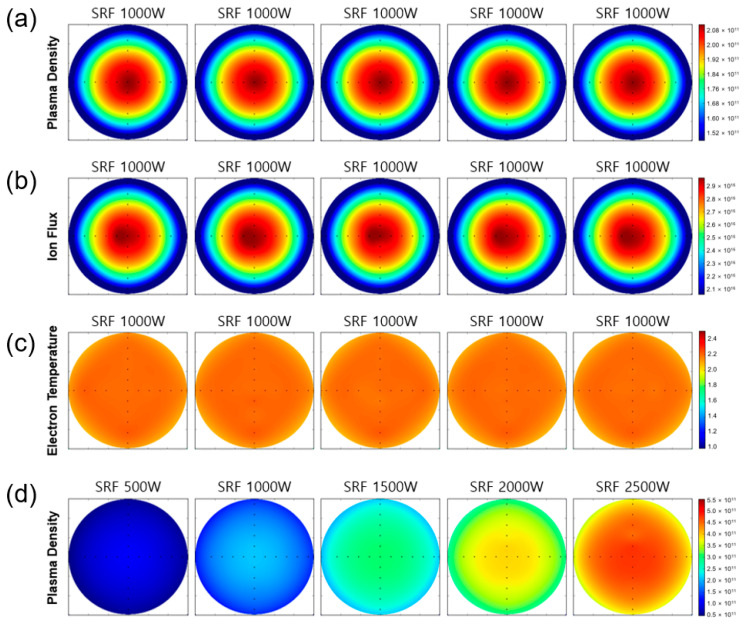
Data integrity and reproducibility of (**a**) plasma density, (**b**) ion flux, and (**c**) electron temperature of an argon gas at an SRF of 1000 W. (**d**) Linearity performance of a plasma density while increasing the SRF power (i.e., 500 W~2500 W).

As illustrated in Figure 10a,b, the proposed sensor showed superior data reproducibility during the repetitive monitoring of argon plasma under the same chamber condition. The resulting value of plasma density (i.e., 10^11^~10^12^ cm^−3^), ion flux (i.e., 10^16^~10^17^ cm^−2^·s^−1^), and electron temperature (i.e., ~2 eV) of argon plasma were in good agreement with theoretical values [19,22,23]. In addition, the proposed sensor exhibited a good system performance in terms of linearity of plasma density after increasing the SRF power from 500 W to 2500 W (Figure 10d). We note that the ion flux of argon plasma also showed a similar tendency to that of plasma density (Figure 10d) after a step-wise increase of the SRF power from 500 W to 2500 W.

In Figure 10d, an anomaly-like point can be seen at one of the probe units (i.e., 12 o’clock direction, the fourth one counting from the wafer edge) in plasma density. One possibility of this anomaly may correspond to the instantaneous plasma turbulence during the plasma processing, such as micro-arcing. To minimize the undesirable local errors that may occur during plasma diagnostics, we further implemented an algorithmic “averaging” function into the proposed sensor. This function allows for multiple averaging of plasma data over a given sampling period and returns the average value of the processed data.

## 4. Summary and Discussion

In this work, we present a newly developed wafer-type in situ plasma monitoring sensor that features an ultra-thin system thickness of ~1.4 mm. As far as we know, it is the thinnest realization among all wafer-type plasma diagnostic tools. In order to achieve the above breakthroughs, we have incorporated our engineering know-how and techniques, including not only a probe design and advanced wafer processing and silicon bonding that allowed for the robust mechanical design of an ultra-thin wafer-type sensor but also a low-power analog and digital circuit designs and innovative signal processing algorithms that dramatically reduced both power consumption and computational burden in the proposed sensor. The development of in-house data acquisition software and sensor carrier platform also facilitated the versatile and active demonstration of the proposed sensor.

Throughout this work, we reported the various experimental results regarding the system performance of the proposed sensor, including robot arm transfer capability, vacuum and thermal stress durability, and feasibility for plasma diagnosis in terms of data integrity and reproducibility.

In semiconductor manufacturing, it is crucial to ensure an optimal yield by maintaining the same environment among processing chambers, often referred to as TTTM. In this aspect, our sensor has the great potential to enhance the production efficiency of the semiconductor manufacturing process by dramatically reducing the time and effort required for achieving TTTM. We could also expect that applying our wafer-type plasma monitoring sensor will reduce the time required for the initial setup or joint development process (JDP) of the semiconductor equipment.

Furthermore, by collecting and analyzing data across multiple chambers from different vendors, our proposed sensor can also be useful to comparatively evaluate (or normalize) the processing chambers from various vendors; thus, it can further improve production efficiency by minimizing the yield variations between different processing chambers and reducing the likelihood of defects or errors of devices.

Meanwhile, in aspect of practical use in semiconductor manufacturing, it is worth noting that our developed wafer-type plasma monitoring sensor may serve as a valuable complement to needle-like probes rather than a complete replacement. This is because needle-type probes still have the advantage of being able to collect the plasma information while real production wafers are being processed, and this information can be accumulated and subsequently compared with the plasma information monitored by the wafer-type sensors.

Also, we expect that integration of our proposed sensor into existing semiconductor equipment as a basic component could play a crucial role in enhancing the production efficiency in semiconductor manufacturing, similar to other commercially available probe systems (i.e., ESPion^TM^ and Semion^TM^) [28,29,30]. We further expect that our proposed sensor has a significant potential to facilitate unveiling unconventional plasma conditions in various academic studies [31,32].

The initial version of our wafer-type plasma monitoring sensor employs a total of 21 probes to ensure effective monitoring along the radial direction (i.e., x- and y-axis), leaving gaps in other orientations. This design was confirmed with considerations for reducing chamber maintenance time and enhancing productivity. Our next milestone is to release the next design of our wafer-type sensors in response to the demands of users, considering the comprehensive coverage across all orientations and the increased number of double probes in terms of scalability.

Also, we plan to continue improving the system performance suitable for high-power and high-temperature plasma applications in combination with various processing gases and keep evaluating its applicability by using other processing chambers to further enhance its practical use in a real semiconductor production line.

## Data Availability

The data presented in this study are available on request from the corresponding author. Further technical discussions on the sensor development can be pursued in more detail after signing a nondisclosure agreement (NDA).
